# Association between a single mother family and childhood undervaccination, and mediating effect of household income: a nationwide, prospective birth cohort from the Japan Environment and Children’s Study (JECS)

**DOI:** 10.1186/s12889-022-12511-7

**Published:** 2022-01-17

**Authors:** Hiroyuki Kuroda, Atsushi Goto, Chihiro Kawakami, Kouji Yamamoto, Shuichi Ito, Japan Environment and Children’s Study (JECS) Group, Michihiro Kamijima, Shin Yamazaki, Yukihiro Ohya, Reiko Kishi, Nobuo Yaegashi, Koichi Hashimoto, Zentaro Yamagata, Hidekuni Inadera, Takeo Nakayama, Hiroyasu Iso, Masayuki Shima, Youichi Kurozawa, Narufumi Suganuma, Koichi Kusuhara, Takahiko Katoh

**Affiliations:** 1 Department of Health Data Science, Graduate School of Data Science, Yokohama City University, 22-2 Seto, Kanazawa-ku, Yokohama, Kanagawa 236-0027 Japan; 2 Department of Paediatrics, Graduate School of Medicine, Yokohama City University, 3-9 Fukuura, Kanazawa-ku, Yokohama, Kanagawa 236-0004 Japan; 3 Department of Biostatistics, School of Medicine, Yokohama City University, 3-9 Fukuura, Kanazawa-ku, Yokohama, Kanagawa 236-0004 Japan; 4 National Institute for Environmental Studies, 16-2, Onogawa, Tsukuba, Ibaraki 305-8506 Japan

**Keywords:** Single mother family, Single parent family, Vaccines, Childhood undervaccination, Healthcare disparities, Socioeconomic factors, Public health, Causal mediation analysis, Japan

## Abstract

**Background:**

Although childhood undervaccination among single mother families is a concern for child healthcare, their association is still under debate. This study aimed to investigate the association between maternal marital status and the risk of childhood undervaccination and determine the mediating effect of household income.

**Methods:**

We utilised prospective birth cohort from the Japan Environment and Children’s Study (JECS). Of 104,062 foetal records (children) from 97,413 mothers, 82,462 that included mothers recruited between 2011 and 2014, were analysed. Childhood undervaccination was defined as not having been vaccinated with at least one routine vaccine. A log-binomial regression analysis was used to estimate the risk ratio (RR) for the association between maternal marital status and the risk of childhood undervaccination. A causal mediation analysis was further performed to investigate the proportion of the association mediated by household income.

**Results:**

Among 82,462 children, 3188 and 79,274 had unmarried and married mothers, respectively. Childhood undervaccination was observed in 1053 (33.0%) and 16,901 (21.3%) children of unmarried and married mothers, respectively. Maternal marital status was associated with a higher risk of childhood undervaccination (adjusted risk ratio [aRR], 1.34; 95% confidence interval [CI], 1.27 to 1.41). Compared with married and older mothers, both unmarried and older (aRR, 1.54; 95% CI, 1.35 to 1.77) and unmarried and younger (aRR, 1.66; 95% CI, 1.54 to 1.79) mothers were associated with a higher risk of childhood undervaccination. The causal mediation analysis showed that the proportion mediated by household income was 10.5% (95% CI, 9.9 to 11.0%).

**Conclusions:**

This nationwide, prospective, large-scale birth cohort study found that a household with a single mother was associated with an increased risk of childhood undervaccination, and 10% of this association was explained by household income. These findings underscore the importance of improving the social environment among single mother families, including not only poverty but also working conditions.

**Supplementary Information:**

The online version contains supplementary material available at 10.1186/s12889-022-12511-7.

## Background

Vaccination is a major contributor to improved public health, especially among children, preventing 2–3 million deaths every year [1]. The three-dose completion rate of the diphtheria-tetanus-pertussis combination vaccine is a measure of vaccination coverage and is over 90% in most developed countries [2]. However, the number of inadequately vaccinated people has been increasing in the United States (US) [3] and Japan [4], which promotes the resurgence of vaccine-preventable diseases [5, 6]. Vaccine hesitancy (VH) is defined as the delay in acceptance or the refusal of vaccination, despite available vaccination services [7]. In a survey of the World Health Organisation (WHO) member countries, VH was reported in 93-94% of the countries [8]. The WHO listed VH as one of the ten threats to global health in 2019 [9], and thus, VH and subsequent childhood undervaccination are worldwide concerns.

The association between a single parent family (i.e., parental marital status) and childhood undervaccination has been under examination since the 1980s [10]. A study in the United Kingdom (UK) suggested an association between a single parent family and childhood measles-mumps-rubella (MMR) vaccination coverage [11]. In the US, the maternal marital status of being unmarried was associated with delayed childhood vaccination [12]. However, some studies found no association between a single parent family and up-to-date childhood vaccination [13] or indicated conflicting results [14–16]. These inconsistencies could be due to insufficient sample sizes, study designs that make it difficult to examine causality, such as cross-sectional studies, and examinations of specific vaccines such as MMR vaccine only. Therefore, the true association between a single parent family and childhood undervaccination remains under investigation.

In this study, we investigated the association between maternal marital status at pregnancy and childhood undervaccination at age 2 years. Most recommended vaccines in Japan are administered before age 2 years [17]. Thus, childhood vaccination status at age 2 years can accurately reflect the childhood vaccination status. Previous studies have also used vaccination status at age 2 years as an outcome [12, 15]. We used a directed acyclic graph (DAG) to represent the causal directions between maternal marital status, childhood undervaccination, and their associated factors, and to identify the potential confounding factors [18]. Single mothers in Japan have a high poverty rate despite their high employment rate [19]. Single mothers spend more time providing childcare and less time working than married mothers. They are also more likely to have difficulty obtaining a high-income job, and their household incomes can fall accordingly. However, it is unlikely that household income will dictate whether or not a mother becomes a single mother. Therefore, although most previous studies have treated the household income as a confounding factor [11, 15, 16, 20, 21], we considered household income as a mediating, rather than confounding, factor in the association between maternal marital status and childhood undervaccination. Furthermore, we conducted a causal mediation analysis to examine the extent to which household income explains the association [22].

## Methods

### Study design and participants

This cohort study, which utilised data from the Japan Environment and Children’s Study (JECS), was based on the jecs-ta-20190930 dataset released in October 2019. The JECS is a large-scale, nationwide, multicentre, prospective birth cohort study funded by the Ministry of the Environment of Japan. The detailed study design and baseline characteristics of the JECS cohort were described elsewhere [23, 24]. Mothers in the early pregnancy stage were eligible. Recruitment occurred nationwide between January 2011 and March 2014 in the Study Areas of Japan. Self-administered questionnaires were sent to participants in the first trimester of pregnancy, the second to third trimester, 1 month, 6 months, and 1 year after birth, and until their children turned age 13 years thereafter.

JECS participants who met the following criteria were excluded from this analysis: 1) mothers with stillbirths, miscarriages, or unknown birth outcomes (*n* = 3739); 2) withdrew consent (*n* = 19); 3) at least one questionnaire was not completed (*n* = 15,319); 4) child vaccination data were missing or logically incorrect (*n* = 2205); and 5) maternal marital status data at pregnancy were missing (*n* = 318).

### Measures

The primary outcome was childhood undervaccination at age 2 years. Two types of vaccines are currently available in Japan. One is a routine vaccine that can be administered at no cost to an individual, and the other is a voluntary vaccine that can be administered at the individual’s expense [17]. Among vaccines that are administered before age 2 years, we analysed those against nine pathogens or diseases that were being routinely administered at the time of participants’ recruitment. The nine pathogens or diseases were *Haemophilus influenzae* type b (Hib), *Streptococcus pneumoniae*, diphtheria, pertussis, tetanus, polio, Bacille de Calmette et Guerin (BCG), measles, and rubella. The following vaccine types were categorised into the “vaccinated” group: combination (such as measles-rubella [MR]) and single vaccines; oral live and inactivated polio vaccines; and multiple vaccines that are administered before age 2 years (such as the Hib vaccine), if they were administered at least once. Childhood undervaccination was defined as not having been vaccinated at age 2 years with at least one or more of these vaccines.

The exposure variable, maternal marital status at pregnancy, was obtained from a questionnaire sent in the first trimester of pregnancy and was categorised as being married (defined as being lawfully or de facto married) or unmarried (defined as never been married, divorced, or widowed).

The characteristics of participants included in our analyses were as follows: maternal age at pregnancy, maternal educational level (high school or lower, junior college, or university or higher), annual household income (< 2 million, 2 to 4 million, 4 to 6 million, or ≥ 6 million JPY), maternal job at pregnancy (housewife or unemployed, part-time or self-employed, or full-time), presence of siblings, maternal smoking status during pregnancy, maternal alcohol intake status during pregnancy [25], maternal use of folic acid supplementation during pregnancy, maternal history of vaccine side effects, and maternal mental component summary (MCS) score during pregnancy. The MCS score was calculated based on the Short-Form 8 (SF-8), a shortened version of the SF-36, an internationally used health-related quality of life scale [26, 27].

### Statistical analysis

The DAG was used to represent the causal directions among factors and to identify potential confounding factors [18, 28]. Maternal age at pregnancy (continuous) and maternal educational level (categorical: high school or lower, junior college, or university or higher) were then selected and included in multivariable adjusted models. Since the frequency of outcome was not considered rare, the risk ratio (RR) was estimated by using the log-binomial regression analysis [29]. A subgroup analysis stratified by maternal age and maternal educational level was performed. Combinations of maternal marital status, maternal age, and educational level were also evaluated using the same analysis.

A causal mediation analysis was performed with annual household income as a mediator. Household income was analysed as a continuous variable, with the median income for each category considered as the income for that category. The ≥6 million JPY income category was set to 8 million JPY. The total effect, average causal mediation effect (ACME), average direct effect (ADE), and proportion mediated were estimated [22, 30].

Since the variables used in this study were based on responses to the questionnaires, the occurrence of missing data was predicted. Therefore, multivariate imputation by chained equations (MICE) was performed to create and analyse 200 datasets each for imputing the missing data. The results of analyses using each dataset were combined based on the Rubin’s rule [31]. Analyses using the complete case dataset were also performed for comparison.

Sensitivity analyses were conducted to examine the robustness of the aforementioned analyses. In the sensitivity analysis of the log-binomial regression analysis, E values were calculated for the adjusted RR [32]. That is, the measure of confounding required to fully explain the estimated RR in the presence of uncontrolled confounders was calculated. Since siblings participated in the JECS, some mothers corresponded to more than one child. Children who had the same mother were likely to have similar outcomes, and this had to be considered in the analyses. Therefore, a sensitivity analysis using cluster-robust standard errors and considering the mother as a cluster was conducted [33]. The sensitivity parameter, rho (ρ), which is the correlation between the residuals of the mediator and outcome regressions, was used in the sensitivity analysis for the causal mediation analysis [22, 30, 34]. If there were unadjusted mediator-outcome confounders, the rho value would not be equal to zero [35]. The latter sensitivity analysis was performed using the complete case dataset after confirming that the results of the causal mediation analysis were not largely different between the dataset with missing data imputed and the complete case dataset.

All analyses were conducted using R software (version 4.0.2, R Foundation for Statistical Computing, Vienna, Austria). The package “mice (version 3.11.0)” was used to impute missing data by MICE [36], and the package “mediation (version 4.5.0)” was used for the causal mediation and sensitivity analyses [37]. For all the estimates, 95% confidence intervals (CIs) were calculated and therefore *p*-values of less than 0.05 were considered statistically significant. For the interaction test in the subgroup analysis, p-values of less than 0.05 were also considered significant.

## Results

### Cohort selection and characteristics

**Fig. 1:**
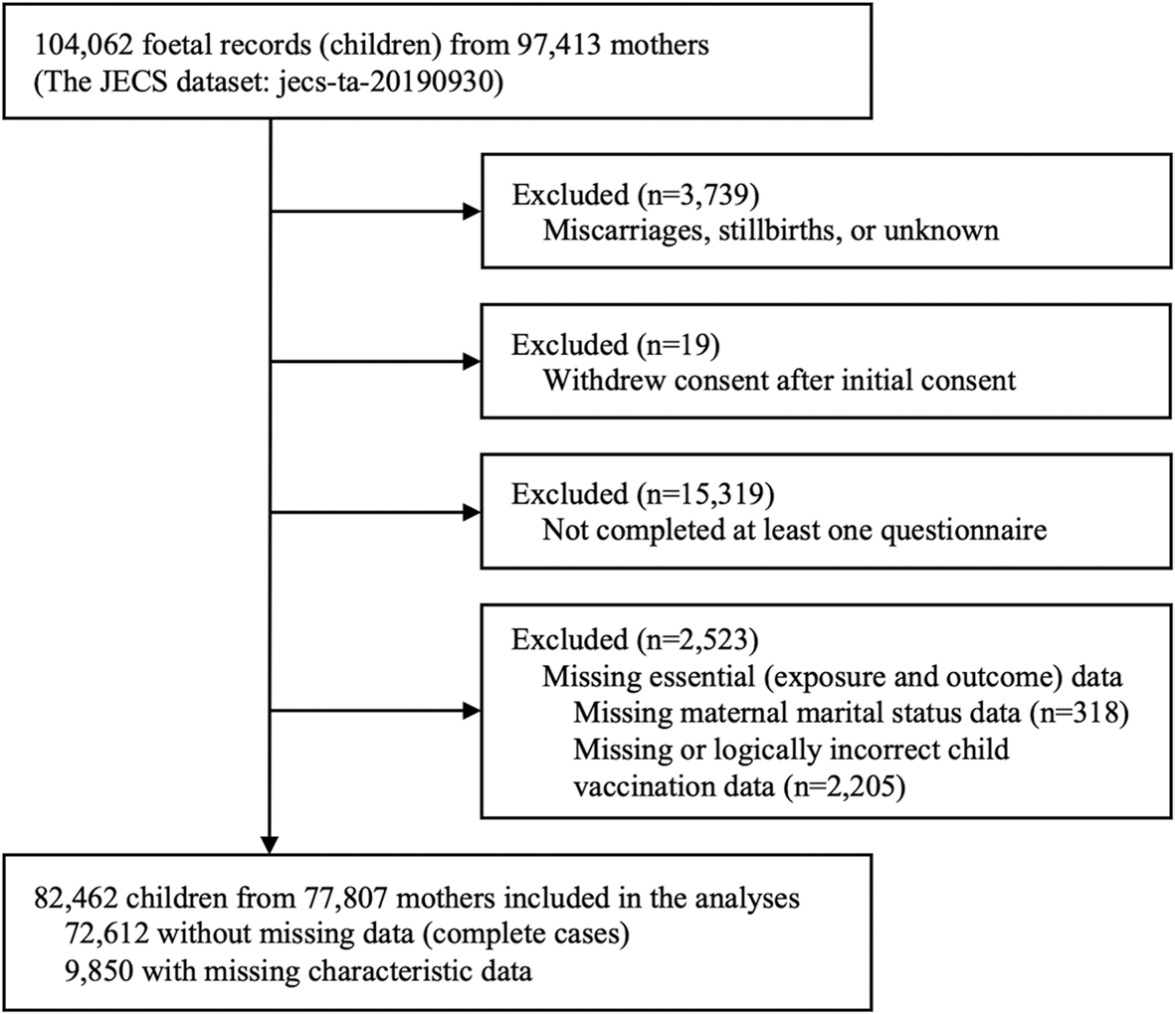
Flow diagram of the cohort selection

Figure 1 shows the flow diagram of the cohort selection. Among the 104,062 foetal records (children) from 97,413 mothers participating in the JECS, 21,600 were excluded, based on the set exclusion criteria. Hence, the remaining 82,462 from 77,807 mothers were included in subsequent analyses. Table 1 summarises the characteristics of the participants stratified by maternal marital status. Of the 82,462 children, 3188 (3.9%) had unmarried mothers and 79,274 (96.1%) had married mothers. Of these children of unmarried and married mothers, 1053 (33.0%) and 16,901 (21.3%) were undervaccinated, respectively. Considering other exploratory comparisons, children of unmarried mothers had younger mothers, lower maternal educational levels, and lower household income than those of married mothers, despite higher employment rates of their mothers. Each vaccine coverage stratified by maternal marital status is shown in Supplementary Table S1.

**Table 1:** Characteristics of participants stratified by maternal marital status (*n* = 82,462)

Variables	Unmarried	Married
	(single, divorced, widowed)	(or de facto marriage)
	*n* = 3188	*n* = 79,274
Maternal age at pregnancy		
Mean (SD)	26.8 (6.1)	31.1 (4.8)
24 or under (%)	1335 (41.9)	6927 (8.7)
25 ~ 29 (%)	859 (26.9)	23,300 (29.4)
30 ~ 34 (%)	577 (18.1)	28,993 (36.6)
35 or over (%)	417 (13.1)	20,050 (25.3)
Data missing (%)	0	4 (0.0)
Maternal educational level (%)		
High school or lower	1932 (60.6)	27,391 (34.6)
Junior college	911 (28.6)	32,967 (41.6)
University or higher	320 (10.0)	18,552 (23.4)
Data missing	25 (0.8)	364 (0.5)
Annual household income (%)		
2 million yen or under	701 (22.0)	3158 (4.0)
2 ~ 4 million yen	1081 (33.9)	25,122 (31.7)
4 ~ 6 million yen	544 (17.1)	25,279 (31.9)
6 million yen or over	361 (11.3)	20,875 (26.3)
Data missing	501 (15.7)	4840 (6.1)
Maternal job at pregnancy (%)		
Housewife or unemployed	865 (27.1)	31,081 (39.2)
Part-time or self-employed	1110 (34.8)	19,403 (24.5)
Full-time	1070 (33.6)	26,316 (33.2)
Data missing	143 (4.5)	2474 (3.1)
Presence of siblings (%)		
Yes	552 (17.3)	43,995 (55.5)
No	2636 (82.7)	35,279 (44.5)
Data missing	0	0
Maternal smoking status during pregnancy (%)		
Yes	382 (12.0)	2688 (3.4)
No	2773 (87.0)	76,103 (96.0)
Data missing	33 (1.0)	292 (0.4)
Maternal alcohol intake status during pregnancy (%)		
Yes	269 (8.4)	8062 (10.2)
No	2903 (91.1)	70,920 (89.5)
Data missing	16 (0.5)	292 (0.4)
Maternal use of folic acid suppl. during pregnancy (%)		
Yes	669 (21.0)	23,479 (29.6)
No	2502 (78.5)	55,610 (70.1)
Data missing	17 (0.5)	185 (0.2)
Maternal history of vaccine side effects (%)		
Yes	48 (1.5)	1009 (1.3)
No	3101 (97.3)	77,821 (98.2)
Data missing	39 (1.2)	185 (0.2)
Maternal MCS score during pregnancy		
Mean (SD)	44.05 (7.57)	46.32 (7.22)
Data missing (%)	80 (2.5)	1286 (1.6)

*suppl.* supplementation, *MCS* mental component summary, *SD* standard deviation

### Log-binomial regression analysis associated with childhood undervaccination

**Fig. 2:**
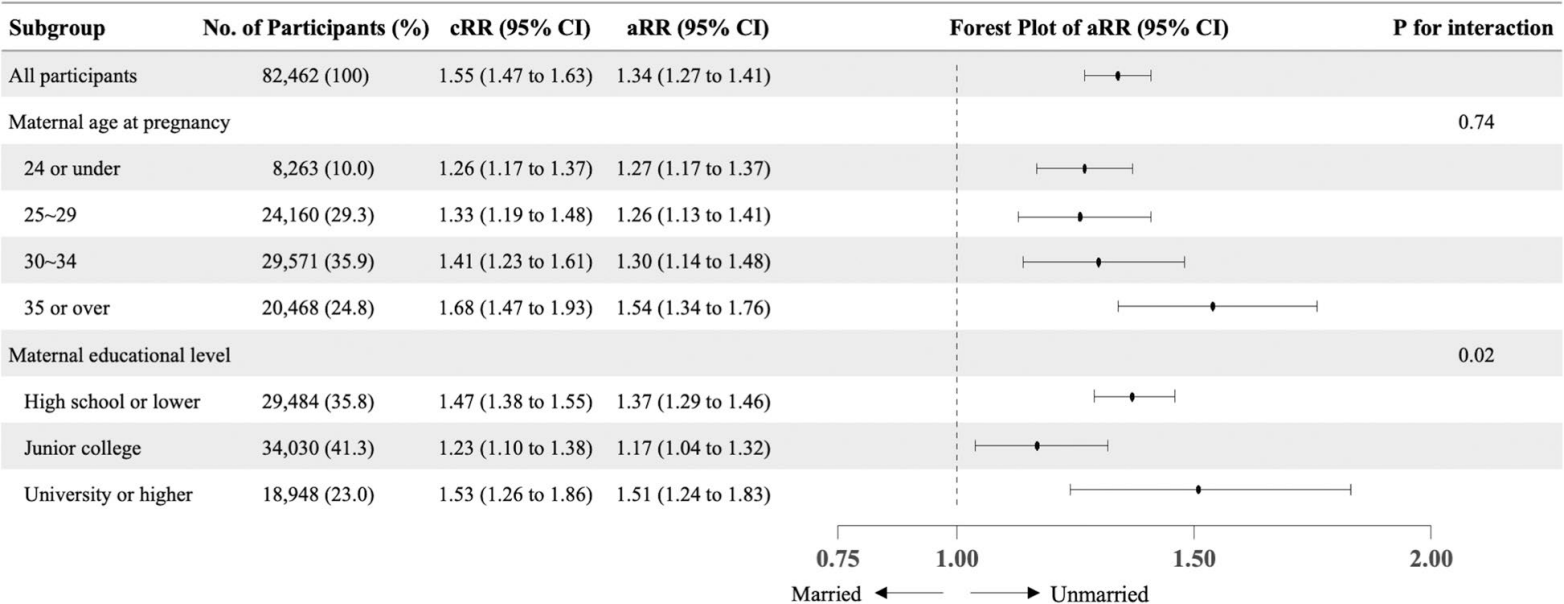
Log-binomial regression analysis including all participants and the subgroup analyses. All estimates and their 95% confidence intervals are risk ratios of unmarried mothers compared to married mothers for childhood undervaccination. The analysis was adjusted for maternal age (continuous) and educational level (categorical). No., Number; CI, confidence interval; cRR, crude risk ratio; aRR, adjusted risk ratio

Supplementary Fig. S1 shows the DAG used in our study. Figure 2 shows the results of the log-binomial regression analysis for all participants and subgroups. After adjusting for maternal age and maternal educational level, maternal marital status was associated with a higher risk of childhood undervaccination (RR, 1.34; 95% CI, 1.27 to 1.41). The subgroup analysis indicated an interaction between maternal marital status and maternal educational level (*P*-value for interaction, 0.02).

**Fig. 3:**
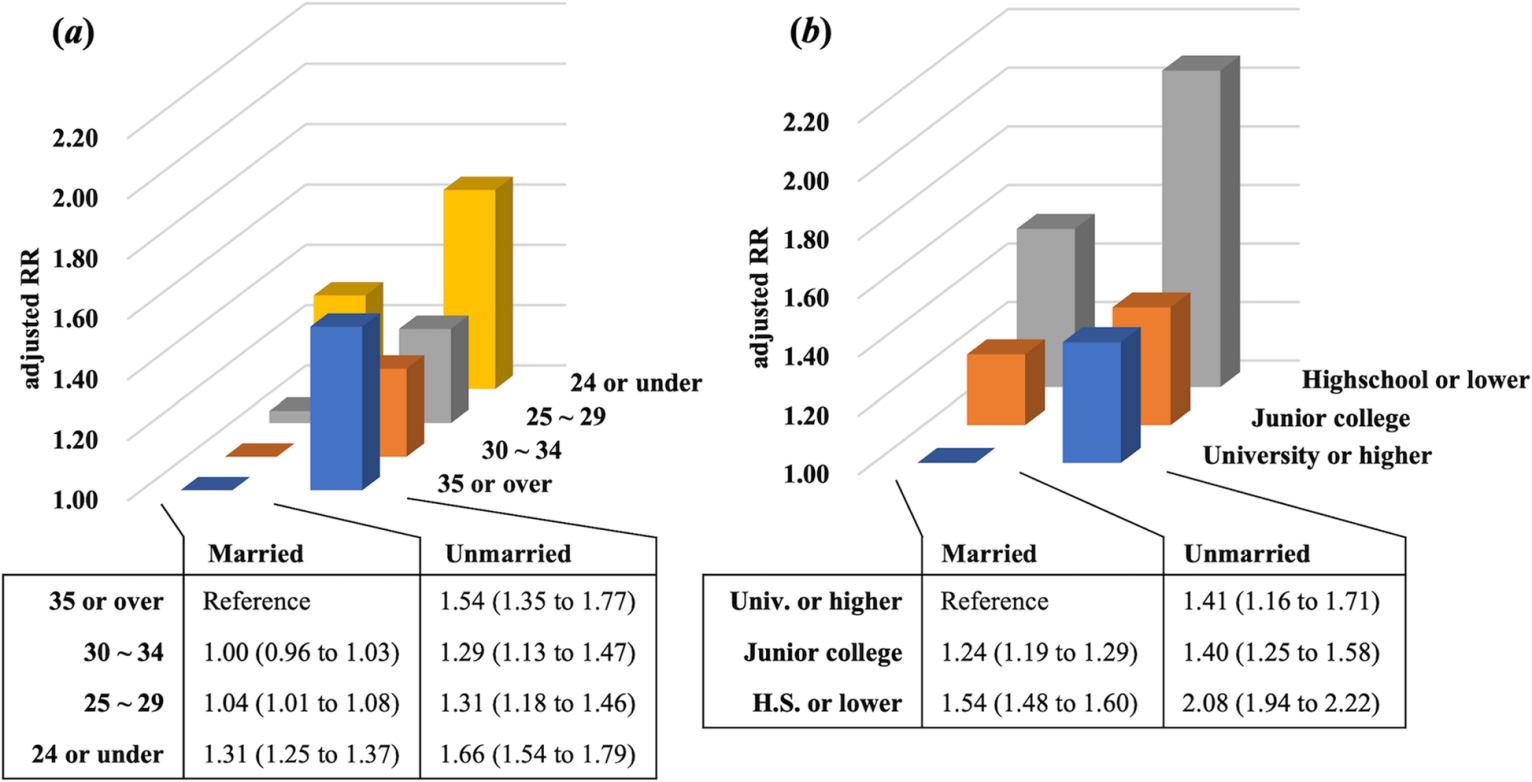
Log-binomial regression analysis using the combinations of maternal marital status and maternal age or educational level. **a** Married mothers aged ≥35 years were the reference group. **b** Married mothers who were university graduate or higher were the reference group. RR, risk ratio; Univ., university; H.S., high school

Figure 3 shows the results of the log-binomial regression analysis using combinations of marital status, age, and educational level. Compared with being married and older (aged ≥35 years), being unmarried and older (RR, 1.54; 95% CI, 1.35 to 1.77) and unmarried and younger (aged ≥24 years) (RR, 1.66; 95% CI, 1.54 to 1.79) among mothers had a higher risk of childhood undervaccination.

### Causal mediation analysis with household income

Table 2 provides the results of the causal mediation analysis with maternal marital status as the exposure, childhood undervaccination as the outcome, and household income as the mediator. Estimates, except for the proportion mediated, were calculated as risk differences. The total effect was 0.0783 (95% CI, 0.0623 to 0.09), the ACME was 0.0082 (95% CI, 0.0068 to 0.01), and the proportion mediated was 0.105 (95% CI, 0.099 to 0.11) in the adjusted model. The results of the analysis using the complete case dataset are presented in Supplementary Table S2.

**Table 2:** Mediation and sensitivity analyses of the mediation effect of household income as the mediator and childhood undervaccination as the outcome

	Crude model	Adjusted model
ACME (95% CI)^a^	0.0242 (0.0217 to 0.03)	0.0082 (0.0068 to 0.01)
ADE (95% CI)^a^	0.0929 (0.0766 to 0.11)	0.0700 (0.0542 to 0.09)
Total effect (95% CI)^a^	0.1171 (0.1006 to 0.13)	0.0783 (0.0623 to 0.09)
Proportion mediated (95% CI)^a^	0.207 (0.200 to 0.21)	0.105 (0.099 to 0.11)
Rho at which ACME = 0^b^	−0.07 to −0.06	−0.06

Estimates are risk differences, except for the proportion mediated

“Rho at which ACME = 0” refers to the sensitivity parameter, rho at which the CI of ACME includes zero. The model was adjusted for maternal age and educational level

*CI* confidence interval, *ACME* average causal mediation effect, *ADE* average direct effect

^a^Calculated with the dataset with missing data imputed (*n* = 82,462)

^b^Calculated with the complete case dataset (*n* = 72,612)

### Sensitivity analyses

The E value for the adjusted RR of unmarried versus married mothers and the lower limit of the 95% CI were 2.01 and 1.86, respectively. The log-binomial regression analysis with cluster-robust standard errors, considering the mother as a cluster, showed that the adjusted RR for childhood undervaccination among unmarried mothers to married mothers was 1.34 (95% CI, 1.27 to 1.41), similar to the main analysis shown in Fig. 2. As shown in Table 2 and Supplementary Table S2, concerning the causal mediation analysis, the results were not significantly different between the dataset with missing data imputed and the complete case dataset. Therefore, we calculated the sensitivity parameter, rho, using the complete case dataset. The rho is also given in Table 2. The rho at which the CI of ACME included zero was − 0.07 to − 0.06 and − 0.06 for the crude and adjusted models, respectively. The rho was slightly closer to zero when maternal age and maternal educational level were added as covariates.

## Discussion

This nationwide, prospective birth cohort study investigated the association between maternal marital status and childhood undervaccination at age 2 years. Unmarried mothers were approximately 1.3 times more likely to have childhood undervaccination than married mothers. Furthermore, although the social backgrounds of younger and older unmarried mothers may be different, our findings suggest that both are at risk of childhood undervaccination. We subsequently conducted the causal mediation analysis using household income as the mediator. Approximately 10% of the association between maternal marital status and childhood undervaccination was explained by household income.

Clarifying the association between the two can provide an evidence-based justification for social support. The vaccines surveyed in this study were provided at no cost to the individuals. The result of the mediation analysis, which showed that the mediating effect of household income was low, was consistent. Another possible barrier to childhood vaccination is the lack of time for child healthcare among households with single mothers. Compared to the Organisation for Economic Co-operation and Development (OECD) countries, single mothers in Japan have higher poverty rates despite higher employment rates [19]. Our study similarly showed that unmarried mothers had higher employment rates and lower household incomes than married mothers. To solve this problem, improvement in the working conditions among single mothers is needed. Furthermore, for example, simplification of vaccination systems, institutionalisation of paid leave for child healthcare, and vaccination programs at the mother’s workplace can facilitate access to child vaccination for single mothers who are busy with both home and work. We not only investigated the mediating effect of household income but also highlighted the problem of managing work and child healthcare among single mothers.

Supplementary Table S1 shows that the vaccine coverage after age 1 year, such as for measles, was less than that of the vaccines given in early infancy, such as pneumococcus. This suggested that some children were vaccinated in early infancy but gradually dropped out. In contrast, some families did not vaccinate their children at all. Of the children of unmarried and married mothers, only 12/3188 (0.4%) and 191/79,274 (0.2%), respectively, were not vaccinated at all. The background characteristics of families who gradually dropped out and did not vaccinate their children at all may be different. However, we could not compare the characteristics between the two groups because of the small sample size of families who were not vaccinated at all.

### Comparison with other studies

Several previous studies had similar findings. A US cross-sectional study of 14,810 children aged 24 to 35 months suggested an association between maternal marital status and severely delayed childhood vaccinations (adjusted odds ratio, 1.3; 95% CI, 1.1 to 1.6) after adjusting for covariates, including maternal age and maternal educational level [12]. Another UK cohort study of 14,578 children aged 3 years showed that a single parent household was associated with childhood uptake of MMR vaccine (adjusted RR, 1.31; 95% CI, 1.07 to 1.60) [11]. We found a similar association using the larger sample size in our prospective birth cohort data for all recommended vaccines at age 2 years in Japan.

Some previous studies have also examined the association between household income and childhood vaccination status. However, regardless of the timing of the studies, they did not find an association between the two. A US study of 4431 children conducted in 1994 indicated no association between household income and up-to-date childhood vaccination [38]. Another Canadian study of 3604 children conducted in 2013 suggested that household income and childhood uptake of measles vaccine did not show a clear trend [15]. These studies did not find an association between household income and childhood vaccination because of lack of power due to the relatively small sample sizes. Otherwise, these results could be evidence suggesting that household income should be considered as a mediating factor for a causally higher-level factor (i.e., maternal marital status examined in our study). We conducted a causal mediation analysis based on this hypothesis.

### Strengths and limitations

The key strength of our study was its large-scale and prospective birth cohort data. Previous studies were mostly designed as cross-sectional studies [39]. Our study design is more suitable for investigating causal associations. We followed children and their mothers until the children turned 2 years old. Since most vaccines are administered in infancy, up to 1 year old, childhood undervaccination may have occurred already by this time. A previous study showed an association between childhood vaccination status at 1 year old and maternal marital status [40]. Therefore, the follow-up period from early pregnancy until the children turn age 2 years is sufficient to estimate the causal association between the two.

Our study had some limitations. First, the characteristics of the JECS cohort may not accurately reflect those of Japanese pregnant women. The JECS participants were recruited through Co-operating health care providers and government agencies. Mothers who have negative views on vaccination (i.e., those with VH) may avoid visiting them. Hence, the JECS cohort may have underestimated the frequency of VH. However, the JECS recruited sufficient participants nationwide. Since the coverage for each vaccine was not largely different from those reported previously [41], the effect of this limitation might be negligible. In addition, the enrolled participants were mothers, and single fathers were not included in this study. Since single mothers and single fathers can have different socioeconomic conditions, the results of this study may not be generalisable to single fathers. Second, variables may have been misclassified due to the self-reported JECS surveys. In our study, unmarried mothers had a lower socioeconomic status than married mothers. Thus, unmarried mothers may be more likely to give responses that differ from the true vaccination status of their children. Misclassification of outcomes may be more likely among unmarried mothers, causing a differential misclassification. Moreover, the maternal marital status at pregnancy can change during the study. Some mothers may marry after childbirth, and others may divorce. Biases can occur if the exposure factor (marital status) changes during the period up to the occurrence of the outcome (childhood vaccination). Third, our analyses may have unmeasured residual confounding. Therefore, we conducted sensitivity analyses using the E value and the sensitivity parameter, rho. The E value was higher than the RRs for the other measured variables and the rho was close to zero. Thus, it is unlikely that unmeasured confounders would completely account for the observed association and explain the estimated mediating effect. Furthermore, the causal mediation analysis in this study was conducted only with household income. As shown in Supplementary Fig. S1, several factors, such as maternal employment status and living with grandparents, were considered as mediating factors in the association between maternal marital status and childhood undervaccination. In order to examine effective support for single mothers, analysing the mediating effects of factors other than household income is also needed.

### How to improve childhood vaccination coverage in single mother families

Poverty in single mother families as well as VH are serious problems. The average poverty rate in OECD countries, including Japan, has been 9.8% for families with two parents. In contrast, for single mother families, it has been 32.5% [42]. Although the “welfare-to-work” interventions have been attempted to improve employment status and single mother family incomes, the effects are limited [43]. Moreover, single mother families in Japan have the lowest incomes among OECD countries despite their high employment rates [19].

Vaccination system issues, such as supply and geographical accessibility, have been identified as reasons for undervaccination in low and middle-income countries [44]. In contrast, among single mothers in Japan and other developed countries, lack of time for child healthcare due to busy work, including housework, can contribute to undervaccination. The high rates of employment and poverty among single mothers in Japan indicate their busyness. Both financial support and comprehensive improvement in the socioeconomic status of single mother families are needed. Moreover, being a single mother is not in itself a modifiable risk factor. Instead, the causal mediation analysis conducted in our study can help investigate interventions that are effective for improving childhood vaccination coverage of single mother families.

## Conclusions

We investigated the association between maternal marital status and childhood vaccination status in a Japanese nationwide birth cohort. Furthermore, we examined the mediating effect of household income on this association. When mothers were unmarried, childhood undervaccination increased by approximately 1.3 times. Moreover, 10% of this association was explained by household income. Our study suggests both an effect of financial support and the problem of balancing work and child healthcare among single mothers. The focus needs to be directed towards the main problems of maternal working conditions and poverty.

## Supplementary Information

**Additional file 1.**

**Additional file 2.**

**Additional file 3.**

## Data Availability

Data are unsuitable for public deposition because of the ethical restrictions and legal framework of Japan. It is prohibited by the Act on the Protection of Personal Information (Act No. 57 of 30 May 2003 amendment on 9 September 2015) to publicly deposit data containing personal information. Ethical Guidelines for Medical and Health Research Involving Human Subjects enforced by the Japan Ministry of Education, Culture, Sports, Science and Technology and the Ministry of Health, Labour and Welfare also restrict the open sharing of epidemiologic data. All inquiries about access to data were sent to: jecs-en@nies.go.jp. The person responsible for handling enquiries sent to this e-mail address is Dr. Shoji F. Nakayama, JECS Programme Office, National Institute for Environmental Studies.

## Abbreviations

US: United States
VH: Vaccine hesitancy
WHO: World Health Organisation
UK: United Kingdom
MMR: Measles-mumps-rubella
DAG: Directed acyclic graph
JECS: Japan Environment and Children’s Study
Hib: *Haemophilus influenzae* type b
BCG: Bacille de Calmette et Guerin
MR: Measles-rubella
MCS: Mental component summary
SF-8: Short-Form 8
RR: Risk ratio
ACME: Average causal mediation effect
ADE: Average direct effect
MICE: Multivariate imputation by chained equations
CI: Confidence interval
OECD: Organisation for Economic Co-operation and Development
