# Supplementary figures and images for: Association between a single mother family and childhood undervaccination, and mediating effect of household income: a nationwide, prospective birth cohort from the Japan Environment and Children’s Study (JECS)
Source: BMC Public Health. 2022 Jan 17;22:117. doi: 10.1186/s12889-022-12511-7 (PMC8764848; doi:10.1186/s12889-022-12511-7)

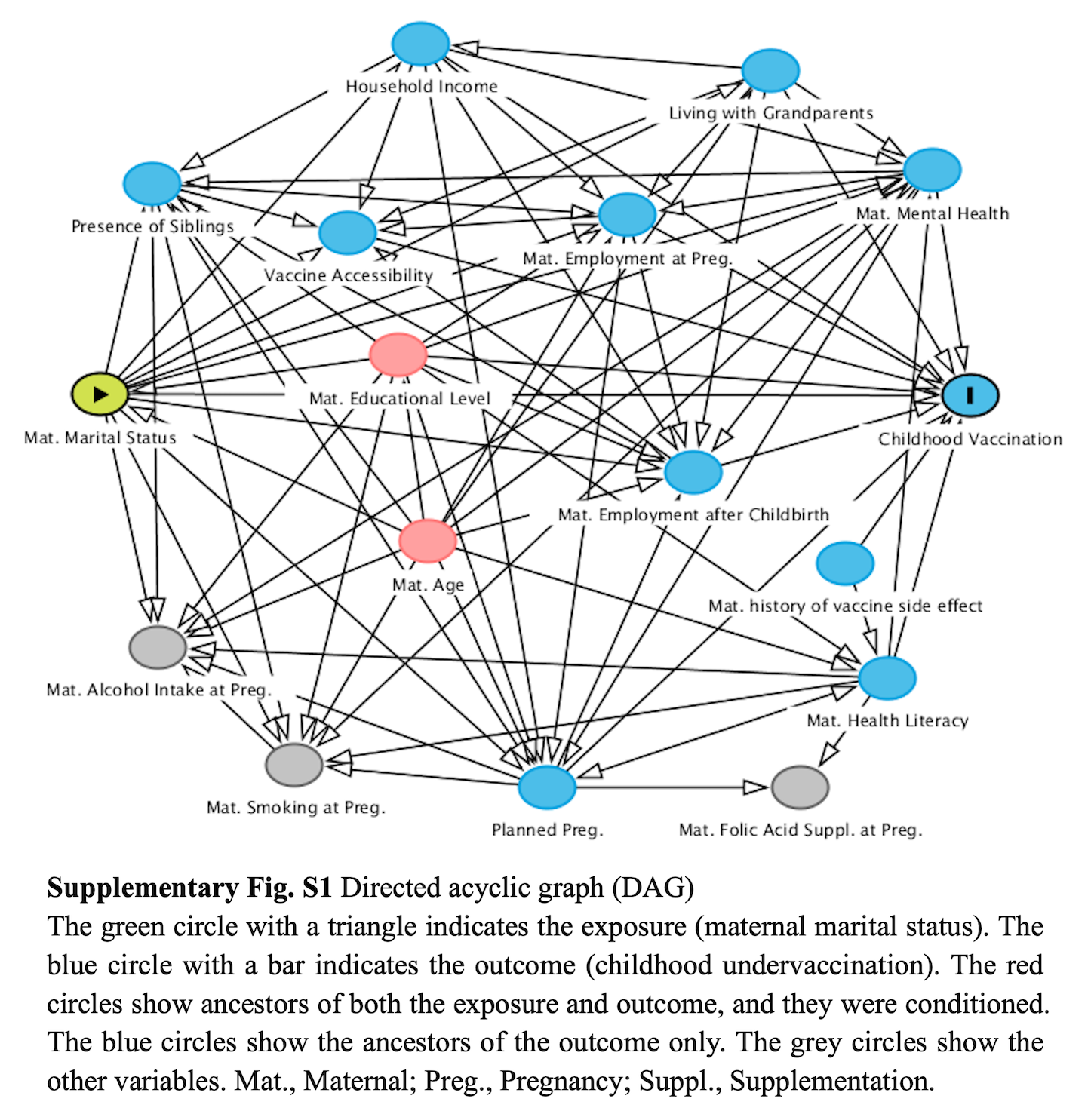

Supplement: Supplementary file 1 — Additional file 1. [file 12889_2022_12511_MOESM1_ESM.tiff]
