# Supplementary material for: Association between a single mother family and childhood undervaccination, and mediating effect of household income: a nationwide, prospective birth cohort from the Japan Environment and Children’s Study (JECS)
Source: BMC Public Health. 2022 Jan 17;22:117. doi: 10.1186/s12889-022-12511-7 (PMC8764848; doi:10.1186/s12889-022-12511-7)
Supplement: Supplementary file 2 — Additional file 2. [file 12889_2022_12511_MOESM2_ESM.docx]

| **Supplementary Table S1.** Each vaccine coverage stratified by maternal marital status (n=82,462) | | | |
| --- | --- | --- | --- |
|  | **Unmarried** | **Married** |  |
|  | (single, divorced, widowed) | (or de facto marriage) | p-values |
| **Vaccines (%)** | **n=3,188** | **n=79,274** |  |
| Hib | 2,885 (90.5) | 74,030 (93.4) | <0.001 |
| S.pneumoniae | 2,910 (90.5) | 73,803 (93.1) | <0.001 |
| Diphtheria | 3,144 (98.6) | 78,544 (99.1) | 0.011 |
| Pertussis | 3,144 (98.6) | 78,542 (99.1) | 0.012 |
| Tetanus | 3,144 (98.6) | 78,544 (99.1) | 0.011 |
| Polio | 3,089 (96.9) | 77,822 (98.2) | <0.001 |
| BCG | 3,080 (96.6) | 77,433 (97.7) | <0.001 |
| Measles | 2,725 (85.5) | 72,694 (91.7) | <0.001 |
| Rubella | 2,534 (79.5) | 70,160 (88.5) | <0.001 |
| P-values are calculated by chi-squared test. Hib, *Haemophilus influenzae* type b; S.pneumoniae, *Streptococcus pneumoniae*; BCG, Bacille de Calmette et Guerin. | | | |
