# Supplementary material for: Association between a single mother family and childhood undervaccination, and mediating effect of household income: a nationwide, prospective birth cohort from the Japan Environment and Children’s Study (JECS)
Source: BMC Public Health. 2022 Jan 17;22:117. doi: 10.1186/s12889-022-12511-7 (PMC8764848; doi:10.1186/s12889-022-12511-7)
Supplement: Supplementary file 3 — Additional file 3. [file 12889_2022_12511_MOESM3_ESM.docx]

| **Supplementary Table S2.** Mediation and sensitivity analyses of the mediation effect of household income as the mediator and childhood undervaccination as the outcome, using the complete case dataset (n=72,612) | | |
| --- | --- | --- |
|  | Crude model | Adjusted model |
| ACME (95% CI) | 0.0084 (0.0065 to 0.01) | 0.0085 (0.0071 to 0.01) |
| ADE (95% CI) | 0.0614 (0.0444 to 0.08) | 0.0631 (0.0477 to 0.08) |
| Total effect (95% CI) | 0.0698 (0.0525 to 0.09) | 0.0716 (0.0565 to 0.09) |
| Proportion mediated (95% CI) | 0.123 (0.082 to 0.17) | 0.118 (0.088 to 0.17) |
| Rho at which ACME = 0 | -0.07 to -0.06 | -0.06 |
| Estimates are risk differences, except for the proportion mediated. CI, confidence interval; ACME, average causal mediation effect; ADE, average direct effect. “Rho at which ACME = 0” refers to the sensitivity parameter, rho at which the CI of ACME includes zero. The model was adjusted for maternal age and educational level. | | |
